# Necrotizing Fasciitis—Severe Complication of Bullous Pemphigoid: A Systematic Review, Risk Factors, and Treatment Challenges

**DOI:** 10.3390/medicina59040745

**Published:** 2023-04-11

**Authors:** Milan Stojičić, Milana Jurišić, Milana Marinković, Miodrag Karamarković, Milan Jovanović, Jelena Jeremić, Marko Jović, Aleksandar Vlahović, Mladen Jovanović, Kristina Radenović, Nikola Jovićević, Dolika Vasović

**Affiliations:** 1 Clinic for Burns, Plastic and Reconstructive Surgery, University Clinical Center of Serbia, 11000 Belgrade, Serbia; 2 Faculty of Medicine, University of Belgrade, 11000 Belgrade, Serbia; 3 Institute for Mother and Child Health Care of Serbia, 11000 Belgrade, Serbia; 4 Clinic for Plastic and Reconstructive Surgery, Clinical Center of Vojvodina, 21000 Novi Sad, Serbia; 5 Faculty of Medicine, University of Novi Sad, 21000 Novi Sad, Serbia; 6 Clinic for Neurosurgery, University Clinical Center of Serbia, 11000 Belgrade, Serbia; 7 Clinic for Eye Diseases, University Clinical Center of Serbia, 11000 Belgrade, Serbia

**Keywords:** necrotizing soft tissue infection, LRINEC score, bullous pemphigoid, autoimmune skin blistering disease, immunosuppression

## Abstract

*Background and objectives:* Bullous pemphigoid (BP), the most common subepidermal autoimmune skin blistering disease (AIBD) has an estimated annual incidence of 2.4 to 42.8 new cases per million in different populations, designating it an orphan disease. Characterized by disruption of the skin barrier combined with therapy-induced immunosuppression, BP could pose a risk for skin and soft tissue infections (SSTI). Necrotizing fasciitis (NF) is a rare necrotizing skin and soft tissue infection, with a prevalence of 0.40 cases per 100,000 to 15.5 cases per 100,000 population, often associated with immunosuppression. Low incidences of NF and BP classify them both as rare diseases, possibly contributing to the false inability of making a significant correlation between the two. Here, we present a systematic review of the existing literature related to the ways these two diseases correlate. *Materials and methods:* This systematic review was conducted according to the PRISMA guidelines. The literature review was conducted using PubMed (MEDLINE), Google Scholar, and SCOPUS databases. The primary outcome was prevalence of NF in BP patients, while the secondary outcome was prevalence and mortality of SSTI in BP patients. Due to the scarcity of data, case reports were also included. *Results:* A total of 13 studies were included, six case reports of BP complicated by NF with six retrospective studies and one randomized multicenter trial of SSTIs in BP patients. *Conclusions:* Loss of skin integrity, immunosuppressive therapy, and comorbidities commonly related to BP patients are risk factors for necrotizing fasciitis. Evidence of their significant correlation is emerging, and further studies are deemed necessary for the development of BP-specific diagnostic and treatment protocols.

## 1. Introduction

Bullous pemphigoid (BP), the most common subepidermal autoimmune skin blistering disease (AIBD), has an estimated annual incidence of 2.4 to 42.8 new cases per million in different populations, designating it an orphan disease [1,2,3,4,5,6]. The circulating autoantibodies in bullous pemphigoid are targeted against epidermal proteins in the hemidesmosomal antigens BP180 and BP230, causing blistering at the dermal-epidermal junction [7]. Research has shown a steady increase in the incidence of bullous pemphigoid, while one group of authors found an average yearly increase in the incidence of 17% [1,8]. Bullous pemphigoid patients are found to have a genetic predisposition; still, environmental factors play an important role in disease development, such as viral infections, previous surgical procedures, trauma history, and the patient’s history of medications and vaccinations [9,10,11]. Drug-related bullous pemphigoid can manifest as a drug-induced or drug-triggered disease, usually affecting the younger population. While the cessation of medication use brings a resolution of the disease in drug-induced BP, in drug-triggered BP that is not the case [12]. Beta-blockers, diuretics, antibiotics, non-steroidal anti-inflammatory drugs (NSAIDs), neuroleptics, immune checkpoint inhibitors, as well as anti-tumor necrosis factor (TNF)-α, are described as triggers [13]. One recent study found that 6.28% of the world population, corresponding to 462 million individuals globally, are affected by diabetes, putting an especially heavy weight on the use of dipeptidyl peptidase 4 inhibitors (DPP-4) in diabetic patients’ therapy [14]. The use of DPP-4 inhibitors was found to be associated with a significant increase in the risk of developing BP by a group of authors in one case-control study with an adjusted odds ratio [aOR] of 1.58 and 95% CI, 1.25–2.00 [15]. Moreover, vaccine-induced BP has been previously described, with reports of COVID-19 vaccine-induced bullous pemphigoid cases emerging in the literature with both new onset as well as reactivation cases of BP. Mechanisms behind these findings are still unknown, while some authors indicate molecular mimicry or the activation of B and T cell immunity as the trigger in genetically predisposed individuals [16,17]. Consequently, the healthcare system of the future could expect a further increase in the number of BP patients, with autoimmune skin blistering diseases presenting a major healthcare burden and deserving more public concern.

One of the simplest classifications of disease severity depending on the total body surface area (TBSA) affected describes BP as mild (<10% TBSA), moderate (10 to 30% TBSA), or severe (>30% TBSA) [18]. Therapy for BP depends on the severity of the disease and relies mostly on long-term systemic and topical corticosteroids as well as other immunosuppressive agents [7,18]. In BP patients, given the disruption of the skin as the body’s main barrier, the finding of NF in individuals even after minor events such as abrasions and lacerations seems to place BP patients as hypothetically ideal candidates for such infections. Most studies addressing specific causes of death in BP patients found skin and soft tissue infections (SSTIs) to be one of the most common causes of death, and, additionally, that SSTIs affect virtually all BP patients to some extent.

Necrotizing fasciitis (NF) is a rare necrotizing skin and soft tissue infection (NSTI) with a prevalence of 0.40 cases per 100,000 to 15.5 cases per 100,000 population in the literature [19,20]. NF occurs more often in elderly patients with comorbidities such as diabetes mellitus and chronic renal failure [21]. Other risk factors include skin trauma, immunosuppression, immunosenescence, malnutrition, obesity, alcohol or drug abuse, and peripheral vascular disease. Causes of immunocompromise implicated include iatrogenic (therapy-related) or disease-related immunodeficiency [22,23]. Cases of NF were most commonly described after surgery, as well as spontaneous, without previous trauma. Most importantly, minor events such as tears, abrasions, lacerations, or insect bites as well as routine obstetrical and gynecologic procedures were all found to be associated with necrotizing fasciitis [24,25]. The main characteristic which defines necrotizing fasciitis and differentiates it from other SSTIs is its massive tissue destruction, followed by severe systemic toxicity, hemodynamic collapse, further organ failure, and ultimately, due to its rapid progression, high mortality [24,25].

Such infections in these conditions haven’t been systematically studied in the literature. Low incidences of NF and BP classify them both as rare diseases, possibly contributing to the false inability of making a significant correlation between the two, even though BP patients share an abundance of risk factors for NSTI, discussed in this review. As necrotizing fasciitis itself presents an often belatedly diagnosed, difficult to treat, and perilous soft tissue infection, it is plausible that such complications in BP patients are underreported and the risk of NF in these patients is underestimated [26]. Here, we present a systematic review of the existing literature related to the topic and the ways two diseases have a strong predilection of being highly correlated.

## 2. Materials and Methods

In this systematic review, the aim was to identify studies reporting the incidence, prevalence, and mortality of NF in patients with BP from the year 2000 onward. The systematic review was conducted according to the Preferred Reporting Items for Systematic Review and Meta-Analyses (PRISMA) guidelines [27]. The research was conducted by authors from 5 January 2022 to 15 December 2022. PubMed (MEDLINE), Google Scholar, and SCOPUS databases were searched for the studies including terms: [bullous pemphigoid OR autoimmune bullous disease] AND [necrotizing fasciitis OR skin and soft tissue infections] AND [mortality] used as keywords in various combinations. Publications were limited to English and French. The primary outcome was the prevalence of NF in BP patients, while the secondary outcome was the prevalence and mortality of SSTI in BP patients. Inclusion criteria were human studies addressing skin and soft tissue infections or NF in BP patients. Due to the scarcity of data, case reports were also included if they met the JBI Manual for Evidence Synthesis criteria [28]. Case reports and studies are presented in Table 1 and Table 2, separately.

Two stages of the systematic review were conducted by two teams independently. The first team searched selected terms by title and abstract. Studies not reporting primary data were excluded; however, references were analyzed for additional eligible original studies. 

Abstracts evaluated in the initial screen included in this systematic review had BP and NF or SSTI jointly. Abstracts meeting these criteria were eligible for full-text review by the second team. Duplicate studies and those without retrievable full texts were excluded. Regardless of the study design, all full-text studies meeting the inclusion criteria were included. The following information was collected: author, publication year, study design, reporting method, demographic features, therapy, data regarding SSTI or NF events, and risk factors.

During all stages of the screening and data extraction, team disagreements were resolved through consensus and discussion. All conflicts regarding the data entry process were resolved by senior or corresponding authors.

**Figure 1:**
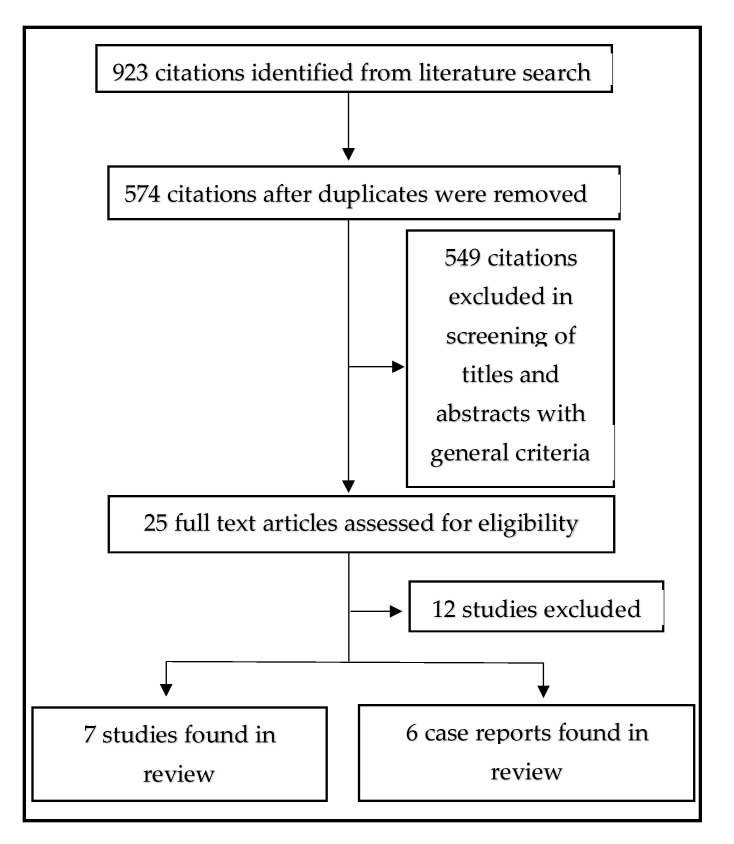
The flow chart of data collection and extraction.

The flow chart of data collection and extraction is presented in Figure 1.

## 3. Results

In our search of BP complicated by NF, six case reports were included with a total of 10 patients, presented in Table 1 [29,30,31,32,33,34,35]. Patients aged from 51–86 years old, with a mean age of 71.5 years. 7/10 patients had a fatal outcome. At the time of NF diagnosis, all patients had incomplete control of the disease. The most common comorbidities described were diabetes mellitus, hypertension, and obesity. Most frequently isolated pathogens were Streptococcus group A (7/10), Methicillin-resistant Staphylococcus aureus (MRSA) (2/10), and Acinetobacter spp. (2/10) with Group A Streptococcus as a monomicrobial source of infections in most cases. 

In cases of diseases with such small incidences, a possible publication bias of case reports could impede the gathering of important clinical events necessary for further investigation. Case reports are frequently discredited as low-evidence reports with a high risk of reporting bias and are rarely published due to their low citability. Still, rare diseases case reports could present the very beginning of high-impact scientific findings such as the correlation of Kaposi sarcoma and HIV in 1981, as well as a more recent finding regarding therapy of infantile hemangiomas with systemic propranolol, both of which began as case reports [42,43].

In the recent literature, 25 studies were found regarding the assessment of infection risk and/or specific causes of death. Studies not providing any information on skin and soft tissue infections (SSTIs) were excluded. The selected studies show conflicting data regarding skin and soft tissue infections, which are shown in Table 2. Most studies addressing specific causes of death in AIBD patients found infections to be the most frequent cause of death while skin and soft tissue infections are found to affect virtually all BP patients [37,38,39,40,41,44,45]. A small retrospective monocentric study by Boughrara et al. investigating infection in patients treated with topical clobetasol propionate (TCP) found that 30% of all participants had any SSTI, with three events of necrotizing fasciitis: one patient with a fatal NF on combination therapy of TCP and mofetil mycophenolate and one patient with fatal NF on combination therapy of TCP and methotrexate. One fatal outcome of NF was reported in a patient on TCP therapy exclusively [30]. Another retrospective study from Mayo Clinic found that 72% of patients studied developed at least one SSTI, and 82% of the cohort developed at least one systemic infection [41]. Although valued for their detailed data regarding infection, the limitations of these studies are their retrospective nature and small patient sample sizes. A study by Cai et al. found skin and soft tissue infections to be the third most common specific cause of death, while Phoon et al. registered only seven events of skin and soft tissue infections [38,46]. Chen et al. found cutaneous infections were one of the two most common complications with 40 SSTIs recorded [41]. A large retrospective study by Ren et al. including a total of 13,342 participants with primary and secondary bullous pemphigoid diagnoses is the only study that found a statistically significant correlation between bullous pemphigoid and diagnosis of necrotizing fasciitis [40]. Considering their incidences, both BP and necrotizing fasciitis have been placed among rare diseases, leaving only a paucity of clinical data for proper investigation and correlation. The large patient sample size of this retrospective study is most likely the contributing factor to eliciting these results. Limitations of all studies are their retrospective nature, bias due to possible differences in hospitalization criteria, and the use of data derived from registers such as death registers and International Classification of Diseases codes in some studies. Skin and soft tissue infections (SSTI) are most commonly divided into two groups: (1) uncomplicated, which affect the superficial layers of the skin and underlying soft tissue, and (2) complicated, necrotizing infections affecting deeper tissues, with systemic effects [26]. Another major factor contributing to reporting bias is clustering infections in one group, such as SSTI. Due to the different severity and outcome of different infections in the same group, this could be potentially misleading, underestimating the prevalence of necrotizing fasciitis in these patients. Not defining the source or the microorganisms in septic patients could also be contributing to reporting bias.

## 4. Discussion

To the best of our knowledge, this is the first systematic review of NF in BP patients.

**Figure 2:**
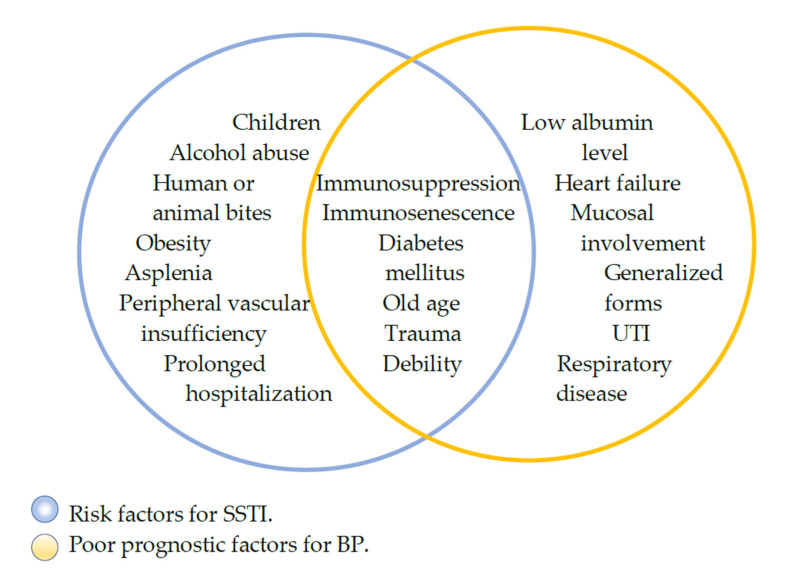
Diagram of shared risk factors for NF and poor outcomes of BP.

Given the disruption of the skin barrier combined with corticosteroid therapy, skin and soft tissue infections are one of the most common complications in these individuals, with impetigo, erysipelas, and cellulitis occurring most frequently [37]. If misdiagnosed or mismanaged, these infections can evolve into complicated, necrotizing infections [25,26]. Complicated, necrotizing soft tissue infections (NSTI) such as necrotizing fasciitis or Fournier’s gangrene commonly cause a systemic inflammatory response or sepsis and tend to rapidly spread into surrounding healthy tissues. Destructive, invasive, and especially fulminant in immunocompromised individuals, they can be fatal with the outcome mainly depending on timely diagnosis and management [47]. Previous studies of other autoimmune diseases such as rheumatoid arthritis, systemic lupus, and scleroderma have proven the major role immunosuppression plays in necrotizing fasciitis, but given the disruption of the skin as the main characteristic of BP and other chronic skin blistering diseases, this BP manifestation as well as comorbidities found in BP patients in the studies included in Table 1 and Table 2, such as diabetes mellitus, chronic renal disease, stroke, Parkinson’s disease, and dementia, could also be major contributing risk factors for NF [48,49,50,51]. Shared risk factors for poor outcomes in patients with bullous pemphigoid, the most common of the autoimmune bullous diseases, and necrotizing fasciitis are shown in Figure 2.

### 4.1. Immunosuppression

Immunosuppression is an important risk factor for necrotizing skin and soft tissue infections and a contributing risk factor for lethal outcomes in BP patients [52]. The risk of infection in patients on systemic immunosuppressant therapy has been immensely researched, mostly following the development of transplant surgery and oncology [53]. Even though significant effort has been made in establishing new therapeutic regimens, systemic corticosteroids remain a cornerstone in BP therapy. They affect both innate and acquired immunity with their effects proven to be dose-dependent; thus, posing the risk of infection in a dose-dependent fashion [54,55]. One retrospective multicentric study by Rzany et al. found prednisolone doses greater than 37 mg/d at discharge a significant risk factor for lethal outcomes in BP patients. Chen et al. also found maximal control dose of corticosteroids a significant contributing factor to skin and soft tissue infection complications, while the same was found by Ren et al. for Cushing’s syndrome due to excessive use of corticosteroids as well as other immunocompromising diseases such as cancer [41,45,56]. A therapeutic milestone study from 2002 by Joly et al. showed that in patients with moderate to extensive disease topical 0.5% clobetasol propionate (TCP) yielded similar therapeutic results as systemic corticosteroids with longer overall survival in the topical corticosteroid group, and minimal to nonsystemic effects [36]. In this randomized controlled trial, in the oral corticosteroid group, one event of necrotizing cellulitis was reported. Concerning treatment, Lehman et al. found that all patients taking oral corticosteroids had a local or systemic infection (100%), while patients on other therapeutic regimens, though still frequently, were less likely to develop any of these infections (88%) [30]. In cases of bullous pemphigoid complicated by necrotizing fasciitis presented in Table 1, 9/10 patients received corticosteroid treatment with one patient’s medical history undisclosed. 5/10 of those patients received combined treatment while 4/10 received corticosteroids exclusively. Moreover, in most cases, the NSTI complication arose in the first month following initiation of BP treatment. Other cytotoxic antineoplastic agents are frequently used in BP therapy such as azathioprine, mofetil mycophenolate, and methotrexate. Working by myelosuppression, inhibition of B and T cell proliferation, and inhibition of antibody production, they are also known to be related to increased risk of infections in cancer patient studies [57]. In recent years, biologic therapy of skin blistering disease patients with rituximab has shown promising results [58,59]. Even though at a lower rate, in these studies infectious complications remained the most frequent adverse event. Necrotizing fasciitis has been a described complication in studies assessing the safety and effectiveness of rituximab therapy in systemic lupus erythematosus as well as in additional case series in recent years [48,49,50]. Keeping that in mind could be of significant importance for clinicians treating these patients while proper data collection and separation of necrotizing fasciitis from other skin and soft tissue infectious complications is deemed necessary for investigators in further studies.

### 4.2. Comorbidities

Diabetes mellitus is the most common underlying condition in necrotizing fasciitis, reported in 44.5% of patients in some studies, and the most frequent comorbidity in patients presented in Table 1 [60]. While in most studies in Table 2 diabetes is one of the most frequent comorbidities found in bullous pemphigoid patients, its influence as a risk factor could be underestimated. Diabetes is one of the most common side effects of corticosteroid therapy with hyperglycemia known to lead to poor wound healing on a cellular, metabolic, and biochemical level [61]. One study found that 40% of all inpatient endocrinology consultations in their hospital are new-onset diabetes cases induced by corticosteroid therapy [62]. This imposes the question of whether this corticosteroid treatment adverse effect is included in the diabetic patient’s group or whether the patients described in BP studies were diagnosed with diabetes prior to hospitalization for bullous pemphigoid, contributing to a certain level of reporting bias. A proper description of inclusion and exclusion criteria for these patients is imperative in assessing the impact of diabetes on bullous pemphigoid patients which would contribute to the development of better NF prevention strategies. Diabetes is a risk factor for uncomplicated skin and soft tissue infections leading to additional subsequent poor wound healing, as well as necrotizing skin and soft tissue infections with a potentially life-threatening outcome [26,63]. Another shared factor for necrotizing soft tissue infections in autoimmune bullous disorders could be neurologic disorders, primarily Parkinson’s disease, dementia, and stroke which would subsequently render patients in need of care and assistance due to debility [38,39,45,46,64]. In these patients, decubitus ulcers or perineal abscesses combined with paraplegia could lead to Fournier’s gangrene or necrotizing fasciitis of the lower abdominal wall [65].

### 4.3. Necrotizing Fasciitis in Immunocompromised Patients

In more recent literature, mortality rates of NF have been reported to be 8.7 to 76% [66]. A study assessing immunocompromised status in necrotizing fasciitis patients found that mortality was significantly higher in immunocompromised than in immunocompetent patients. Most importantly, the first surgical debridement and diagnosis were more often delayed in immunocompromised patients [52]. Urgent diagnosis and surgical debridement are imperative for patient survival. Impediment in treatment is found to increase the chances of fatal outcome 7.5 times in immunocompromised patients, while another study found that mortality rates were 9 times higher if surgical debridement was delayed more than 24 h after the onset of symptoms [67,68]. Necrotizing fasciitis type I is a polymicrobial infection usually described following trauma, caused by aerobe and anaerobe bacteria, most commonly associated with immunocompromised individuals as well as diabetic patients. According to available data, Staphylococcus aureus, β-hemolytic Streptococcus, coagulase-negative Staphylococcus, Escherichia coli, Enterobacter spp, Atropobium parvulum, Morganella morganii, and Klebsiella pneumoniae are the most often isolated bacteria in polymicrobial NF [69]. Type II infection is a monomicrobial infection often developing spontaneously, frequently caused by Streptococcus group A (GAS) and Methicillin-resistant Staphylococcus aureus. The source of infection in these patients is found to be asymptomatic pharyngitis, by systemically seeding as a transient bacteremia [26]. Some studies found that NF caused by gram-negative bacteria was associated with a higher incidence of bloodstream infections and the presence of hemorrhagic bullae, as well as a more rapid disease progression leading to a fatal outcome compared to NF caused by gram-positive bacteria [69].

The most important clinical symptom of necrotizing fasciitis is crescendo pain disproportionate to local findings, while other most common symptoms include local redness and tenderness, soft-tissue edema, and fever [26,65]. The presence of hemorrhagic bullae with bacteremia, purpura, and skin necrosis often follows as signs of advanced disease. Tachycardia, leukocytosis, acidosis, or hyperglycemia combined with the aforementioned local status and the presence of described risk factors should raise high suspicion of necrotizing fasciitis [26,69]. Still, necrotizing fasciitis was found to be misdiagnosed in a mean of 71.4% of 1463 patients by Goh et al. [60]. Several scoring systems have been developed as a diagnostic aide, such as the laboratory risk indicator for necrotizing fasciitis (LRINEC) score based on the following laboratory parameters: white blood cell count, hemoglobin, glucose, creatinine, sodium, and c-reactive protein [70,71]. A major difficulty in early diagnosis of necrotizing fasciitis in immunocompromised individuals is often its altered clinical presentation. Initial infection in these patients can seem subtle, often quickly taking a fulminant course. Due to myelosuppression, laboratory findings can show low white blood cell count, hematocrit, and platelet count. C-reactive protein can present as normal or mildly elevated; thus, compromising the results of LRINEC scoring [52,71]. In hospitalized patients, the use of NSAIDs as antipyretics or analgesics could attenuate pain, further diminishing another clinically important sign. Imaging tests such as MRI or CT scan have proven superior to X-ray, showing edema, fascial thickening, fat stranding, and/or gas in the subcutaneous tissues. Still, surgical debridement should always be given priority with an aggressive antimicrobial therapy covering gram-positive, gram-negative, and anaerobic bacteria administered empirically upon admission following suspicion of necrotizing fasciitis. These patients should be urgently managed by a surgical team experienced in the treatment of immunocompromised patients [25]. 

### 4.4. Surgical Reconstruction of Defects after Management of NF

Reconstruction of defects after necrotizing fasciitis management depends on the size of the defect, localization, the patient’s age, comorbidities, and the availability of healthy tissue. Defects of the abdominal wall were successfully reconstructed by abdominoplasty-type advancement flaps in individual reports, while reports of necrotizing fasciitis affecting the thoracic wall showed successful reconstruction of defects using a meshed bilaminar dermal regeneration template covered with a thin split-thickness skin graft in a patient with scleroderma [51,72]. One systematic review regarding flap coverage after NSTIs found that fasciocutaneous flaps were used most frequently (71%), followed by loco-regional muscle flaps (18%), while free flaps were used less frequently (8%), with rates of partial or complete flap loss of 3.3% [73]. Such reconstructions were performed in patients not eligible for skin grafting, while skin grafting reports are found as individual successful cases in the literature [68,74]. Still, various reports of skin grafting-induced eruptions of bullous pemphigoid on both skin graft and donor sites could be limiting factors for reconstruction of defects in these patients [75,76,77,78,79]. Additionally, in patients with >10 bullae per day or with the Bullous Pemphigoid Disease Area Index (BPDAI) severity score of ≥20 points equaling to a moderate or severe form of BP, in whom remission is not achieved, surgical reconstruction could be limited due to the insufficient healthy skin and soft tissue for flaps or donor sites. Prompt diagnosis and treatment in these patients is imperative for containing the infection from further tissue damage resulting in larger defects. 

### 4.5. Intravenous Immunoglobulin (IVIG)

A small prospective study from 2001 showed all patients with severe BP, affecting 65% to 80% TBSA, obtained and sustained clinical remission receiving IVIG as monotherapy. IVIG was found to have corticosteroid-sparing effects, with no severe side effects [80]. Another more recent randomized double-blind trial found IVIG to reduce disease activity both clinically as well as by lowering anti-BP antibody titers [81]. Conversely, trials regarding IVIG therapy in NF patients showed conflicting results. One randomized control trial of NF patients showed promising results but was prematurely terminated due to slow patient recruitment, while another study found that IVIG was administered to NF patients too infrequently for any statistical significance [82]. Nevertheless, a meta-analysis regarding GAS-associated toxic shock syndrome found a reduction of mortality rates in IVIG-receiving patients after pooling data from studies that previously found no statistical significance independently [83]. 

The mechanism of action of IVIG is still unclear. Some studies of BP patients suggest intravenous immunoglobulins have a significant role in degrading circulating auto-antibodies such as anti–BP180, anti-Dsg1, and anti-Dsg3 IgG, as well as modulating functions of B and T cells and attenuating complement-mediated tissue damage. Whereas in vitro studies regarding IVIG therapy in sepsis found that IVIG preparations hold opsonins and neutralizing antibodies against staphylococcal and streptococcal superantigens and M-protein. So far, group A Streptococcal NF is the only etiology of NF with a clear indication for IVIG [18,81,84,85].

The significance of IVIG in NF patients is still unclear and relies only on a few studies and individual reports in the literature [86,87]. Treatment of NF patients on long-term corticosteroid therapy presents a challenge and tapering or discontinuation of immunosuppressive agents is often necessary. With this in mind, the use of IVIG in critically ill BP patients with NF caused by GAS or Staphylococcus could be justified, combined with aggressive surgical treatment and adequate antimicrobial therapy. 

Side effects of IVIG therapy such as fever, nausea, headache, or malaise are found in about 10% of patients, while severe side effects such as pulmonary edema, thromboembolism, renal failure, and severe anaphylactic reactions are infrequent and are usually observed in patients with IgA deficiency [85]. Regardless of its seemingly many benefits, the use of IVIG is still limited due to its high cost and low availability. 

Currently, ongoing studies are conducted in order to provide safe and effective target therapy for BP patients. Possible targets include CD20+ lymphocytes and interleukin inhibitors as well as inhibitors of the complement system [7]. A fully human monoclonal antibody dupilumab binds to IL-4 receptor and provides significant symptom control, especially in immunocompromised patients such as neoplastic and TBC-infected patients [7,87]. Eosinophils are found to play a crucial role in the pathogenesis of BP, with a positive correlation found between high IgE titers and TBSA affected by BP. Omalizumab, a monoclonal antibody that binds to the Cε3 domain of IgE, as well as bertilimumabin, a monoclonal antibody that targets eotaxin-1, shows promising results [7,88,89]. 

Many protocols have been developed for the management of infections in cancer or transplant patients, but possibly due to low incidences of bullous dermatoses, no protocols have been designed especially for these individuals. Studies of other autoimmune diseases such as systemic lupus erythematosus and rheumatoid arthritis have confirmed that necrotizing fasciitis affects patients on long-term corticosteroid therapy [67,90]. So far, strong evidence correlating BP patients with necrotizing infections relies only on one large study and many other individual reports [45]. Given how timely diagnosis majorly influences survival, larger studies with detailed criteria for defining infections, as well as diabetes and other of the many sharing risk factors are necessary in assessing the correlation between BP and necrotizing fasciitis. Until a clearer picture is drawn, signs of erythema, swelling, crescendo pain, and local warmth together with changes in laboratory parameters should always raise a high suspicion of necrotizing fasciitis in BP patients.

## 5. Conclusions

Loss of skin integrity, immunosuppressive therapy, and comorbidities commonly related to BP patients are risk factors for necrotizing fasciitis. Evidence of their significant correlation is emerging, and further studies are deemed necessary for the development of BP-specific diagnostic and treatment protocols. Signs of erythema, swelling, crescendo pain and local warmth along with changes in laboratory parameters should always raise a high suspicion of necrotizing fasciitis in BP patients.

## Figures and Tables

**Table 1:** A systematic review of all case reports included by the JBI Manual for Evidence Synthesis criteria.

Authors	Age	Sex	Comorbidities	NF Onset (after BP Therapy)	Control of BP	Previous Therapy	Current Therapy	Diagnostics	Bacteriology	NF Treatment	Evolution
Chamberlain 2003 [29]	85	F	HTA	9 d	Incomplete	TCP, Erythromycin 250 mg ×2	Prednisolone 30 mg/g + Betamethasone 500 µg × 2/d mouth wash, Erythromycin 250 mg ×2	Local findings of NF on the left leg, Laboratory, Microbiology, Ultrasound,	Strep A	Successive surgical debridement, split thickness skin grafting Initial antimicrobe therapy: Vancomycin 1 g ×2 Meropenem 1 g ×3 Additional antimicrobial therapy: cefuroxime 1.5 g ×3 clindamycin 300 mg ×4	Survived (sequel)
Boughrara 2010 [30]	73	M	MI, benign vesical polyps	2 years	Incomplete	?	TCP	Local findings of NF in two localizations: left arm and dorsal side of the right hand, Microbiology	Strep A, Pseudomonas sepsis	Debridement of the left arm and amputation of the right index finger Antimicrobial therapy undisclosed.	Died
Boughrara 2010 [30]	64	F	Obesety, insulin dependant DM	14 d	Incomplete	?	TCP, MTX 15 mg/week	Local findings of NF on the left leg, microbiology	Strep A, MRSA, Acinetobacter	Surgical debridement of the dorsal side of the foot and amputation of two toes. Antimicrobial therapy undisclosed.	Died
Boughrara 2010 [30]	81	M	HTA, insulin ndependat DM, preterminal CRI, COPD	14 d	Incomplete	TCP	TCP, MMF	Local finding of NF on left leg and foot, Microbiology	Strep A	Surgical debridement, Antimicrobial therapy undisclosed.	Died
Doffoel-Hantz 2011 [31]	86	F	Acheimer’s dementia	3 weeks	Incomplete	TCP	TCP	Retiform purpura of the ancle followed by development of necrosis.	Strep A	Broad-spectrum antibiotics, no surgical debridement was performed.	Died
Ekiz 2013 [32]	78	F	HTA	3 weeks		Tetracyclin, Niacinamid, TCP, systemic steroids	Prednisolon 48 mg/d after 2 weeks 32 mg/d,	Local finding of NF on the left leg, laboratory, MRI, Microbiology Histopathology	Strep A.	Surgical debridement, Sulbactam/ampicillin 1 g, oral ciprofloxacin 750 mg 2 × 1, tigecycline	Died
Sene 2014 [33]	52	M	Obesity	35 d	Incomplete	TCP	Prednisolone 100 mg/d, after 2 weeks Prednisolone 60 mg/d and MMF 2 mg/d	Local finding of NF on the left leg, laboratory, Microbiology	Strep A	Surgical debridement, pipera- cillin-tazobactam and vancomycin	Survived
Sene 2014 [33]	76	F	HTA, DM	2 weeks	Incomplete	TCP 30 g/d	TCP 10 g/d, MMF 2 g/d	Local finding of NF on the left leg, laboratory, Microbiology	Strep A, MRSA	Surgical debridement piperacillin-tazobactam, gentamicin, and vancomycin	Died
Noguchi 2018 [34]	69	F	DM,	4 d	Incomplete	Prednisolone 20 mg/d, Cyclosporine 100 mg/d	Prednisolone 40 mg/d, IVIG 4 days latter	Local finding of NF on the insertion point of CVC, Laboratory, Microbiology, CT scan	MSSA	Surgical debridement piperacillin-tazobactam and vancomycin	Survived
Jurisic 2023 [35]	51	M	HTA, DM, Obesity	3 weeks	Incomplete	Undisclosed corticosteroid therapy regimen	?	Local finding of deep tissue necrosis due to advanced disease, CT scan, laboratory, microbiology	*Acinetobacter* spp. *Klebsiella-enterobacter* Spp. *Pseudomonas aeruginosa*, *Enterococcus* spp. *Acinetobacter baumannii* complex sepsis (MDR)	Successive surgical debridements, Vancomycin 2 × 1.5 g, Clindamycin 900 mg 3 × 1 and Meropenem 3 × 1 g Additional antimicrobial therapy: Colistimethate-sodium 3 × 3,000,000ij	Died

Abbreviations: Strep A—Streptococcus group A, MRSA—Methicillin-resistant Staphylococcus au reus, MSSA—Methicillin-sensitive Staphylococcus aureus, TCP—topical clobetasol propionate 0.5%, HTA—hypertension, MI—myocardial infarction, MTX—Methotrexate, CRI—chronic renal insufficiency, COPD—Chronic obstructive pulmonary disease, MMF—Mofetil mycophenolate, MRI—magnetic resonance imaging, CVC—central venous catheter, MDR—multi-drug resistant.

**Table 2:** A systematic review of all original articles included.

Author	Year	Type	No. Patients	Mean Age	Therapy	Events	Comorbidities	Risk Factors
Jolly	2002 [36]	Randomized multicenter trial	341	80 ± 11	TCP Oral CS	1 necrotizing Cellulitis in oral CS group	Cardiovascular disease Neurologic disorder DementiaDiabetes mellitus Chronic lung condition	Oral corticosteroid therapy Low Karnofsky score Old age
Boughrara	2010 [30]	Retrospective	30	83.5	TCP MTX MMF	3 NF/10 SSTI	Diabetes Autoimmune diseases	/
Lehman	2013 [37]	Retrospective	54	75.8	TCP Oral CS Cyclosporin MTX MMF Dapsone Rituximab Azathioprine	43 SSTI’s	Diabetes Solid organ cancer/treatment Other autoimmune disorders	Oral corticosteroid therapy
Cai	2014 [38]	Retrospective	359	75.7 ± 2.6	Corticosteroids (88%), Doxycycline and/or nicotinamide (25.9%), Dapsone (13.9%) Azathioprine (39%), Combination therapy of corticosteroids and IMA 37.6%	5 SSTI related causes of death	Heart failure Chronic renal disease Parkinson disease Stroke	Concomitant neurologic disease Heart failure Parkinson disease
Phoon	2015 [39]	Retrospective	97	79 ± 11	Prednisolone + adjuvant therapy in 53% of patients: dapsone 17% doxycycline and nicotinamide 34% azathioprine 3% MMF 6%	7 SSTI’s	Hypertension Neurologic disorders Diabetes	Low Karnofsky score, dementia, higher CCIS
Ren	2018 [40]	Retrospective	13,342	77.3	/	BP was associated with higher odds of necrotizing fasciitis, adjusted OR (95% CI) 2.91 (1.25–6.80), *p* = 0.0136	Rheumatoid arthritis Systemic lupus erythematosus Diabetes Cushing’s Cancer	Older age Higher number of chronic condition Diabetes Cushing’s Cancer
Chen	2020 [41]	Retrospective	252	67.2	Corticosteroids 74.6% Other immunosuppressants 52.0% IVIG 3.6%	40 SSTI’s	Diabetes, Mucosal involvement Respiratory comorbidities	Maximal control dose of corticosteroids, Low serum albumin levels, Hospitalization, Diabetes

TCP—topical clobetasol propionate, CS—corticosteroids, MTX—methotrexate, MMF—Mofetil mycophenolate, NF—necrotizing fasciitis, SSTI—skin and soft tissue infection, IMA—immunomodulatory agents, CCIS—Charlson Comorbidity Index Score, IVIG—intravenous immunoglobulins.

## Data Availability

Not applicable.
